# Cadmium and zinc sorption and desorption in soil: the impact of humic-fulvic acids, *Bacillus* sp., insect frass, and soil aging

**DOI:** 10.1007/s11356-025-36699-4

**Published:** 2025-07-08

**Authors:** Aspasia Grammenou, Georgios Thalassinos, Spyridon A. Petropoulos, Vasileios Antoniadis

**Affiliations:** University of Thessaly, Panepistemio Thessalias, Volos, Greece

**Keywords:** PTEs, Soil remediation, Organic amendments, Sorption mechanisms, Biostimulants

## Abstract

The sorption and desorption of Cd and Zn were investigated in a soil treated and incubated over a period of 30 days with various innovative amendments (the biostimulants humic and fulvic acids (HFA), *Bacillus *sp. (BAC), as well as insect frass (FR), and their combination (FR + HFA and FR + BAC)). This is a novel attempt to study the chemical behavior of the two toxic metals induced by the tested materials—a work that is in high want in the literature. The batch sorption test of Cd and Zn was conducted in the beginning and at the end of the incubation period in competitive systems. We found a significant increase in Cd sorption over time at FR and BAC, as recorded in the distribution coefficient (*K*_d-50_), with values increasing from 143.70 to 184.98 L kg^−1^ and from 144.11 to 196.70 L kg^−1^, respectively. Zn sorption enhanced at FR, with *K*_d-50_ values rising from 940.09 to 1310.64 L kg^−1^. Added frass caused an increase in Cd and Zn sorption relative to the unamended control, but only in day 30, probably due to the added ion exchange and the presence of available organic matter-bound retention sites that contributed to the soil matrix after soil aging over time. Desorption was enhanced for Cd at FR and FR + BAC relative to the control (5.74% and 6.26% vs. 2.08%, respectively), but the fact that no further increase was observed over time is rather promising. We conclude that frass, alone or co-amended with the tested biostimulants, can positively affect metal retention, reducing metal mobility and bioavailability, and thus preserving soil health and environmental safety.

**Supplementary Information:**

The online version contains supplementary material available at 10.1007/s11356-025-36699-4.

## Introduction

Soil contamination by potentially toxic elements (PTEs) represents a significant environmental concern on a global scale (Alam et al. 2024; H. Zhang et al. 2024; Zheng et al. 2024). Among the most prevalent PTEs detected in contaminated soils, cadmium (Cd) and zinc (Zn) play a pivotal role, due to their toxic nature, mobility, and propensity for biomagnification (Grammenou et al. 2024; Ning et al. 2023; Soliman et al. 2024). Industrial activities (such as metallurgy, battery production, and chemical industry), agricultural activities (utilization of phosphate fertilizers), and municipal waste disposal of to soils are the key sources of these elements in the environment (Kikis et al. 2024; Y. Zhang et al. 2023). The presence of these elements may reduce soil fertility and health, adversely affect plant growth and vital ecosystem services, and enter rapidly in food chain (Angon et al. 2024; Soliman et al. 2024). Among them, Cd is well known for its carcinogenic properties (Zulfiqar et al. 2022). On the other hand, Zn, while an essential micronutrient for plants, can be toxic when present in elevated concentrations (Kaur et al. 2024). Therefore, the development of sustainable strategies for reduction and removal of PTEs is necessary.

A variety of methods are utilized for handling and reducing high PTE bioavailability in soil (Golia et al. 2023). Sorption is one of the principal mechanisms of natural depollution, as it controls the fate of PTEs in aquatic and soil environments (Sparks 2005). The general term “sorption” refers to a physicochemical process by which substances attach onto the surface of a solid material (e.g., soil colloids), also known as sorbent, via natural forces or bonds (e.g., electrostatic attraction, hydrogen bonds, van der Waals forces). Desorption, on the other hand, refers to the process by which a previously retained substance is released from the bonding with the sorbent (Chai et al. 2021). The sorption of PTEs and distribution between soil and water are influenced by several key factors. The physical and chemical properties of soil significantly influence PTE fixation, while the chemical specificity of PTEs (e.g., oxidation state, valency, ionic radius) as well as certain chemical properties (e.g., their hydrolysis constant and the degree of the formation of covalent bonds with Si onto the soil colloidal surfaces) plays a crucial role (Ahmed et al. 2022; Antoniadis et al. 2017; Bradl 2004). Also, the concentration of PTEs affects their sorption, as high concentrations lead to the gradual saturation of sorption surfaces, reducing thus the retention ability of adsorbents. However, among many others, soil pH is the single most important factor affecting metal sorption, as it influences almost all other conditions that govern sorption (Bradl 2004; Martínez and Motto, 2000).

It has been demonstrated that the addition of organic amendments to soil can considerably affect the dynamics of metal sorption. Indeed, PTE sorption by soil particles, as well as their desorption, is significantly influenced by soil organic matter (SOM). This can be explained by the fact that SOM contains functional groups, such as carboxylic and phenolic active sites, that are typically deprotonated at normal soil pH, hence with electronegative charge, and thus active to trigger electrostatic attraction towards positive-charged metal cations (Czikkely et al. 2018). Humic acids (HA) and fulvic acids (FA) are naturally occurring organic compounds with multifunctional active groups, such as hydroxyl group (-OH) and carboxyl (-COOH) that contribute to elevate the overall ion exchange capacity of soil. In fact, the oxygen part of these groups contains free electron pairs that form covalent bonds with PTEs, resulting in the chelation and in the creation of stable complexes with PTEs (Ma et al. 2024).

A plethora of microorganisms possesses adaptability in contaminated environments with PTEs, due to the tolerance mechanisms they exhibit. Bacteria classified under the *Bacillus* genus have garnered attention for their crucial role in the remediation of contaminated soils (Alotaibi et al. 2021; Ramírez et al. 2019). Microorganisms have the potential to detoxify and/or degrade PTEs using their physiological and adaptive mechanisms (e.g., efflux, conversion to less toxic form and cytoplasmic accumulation) (Ghosh et al. 2022).

Furthermore, in recent years, there has been a notable increase in scientific interest in insect farming worldwide (Van Huis et al. 2021). Insect larvae have the capacity to convert organic wastes (e.g., crop residues) into high nutritional products, such as proteins, chitins, and lipids (Gkinali et al. 2022). After larvae harvesting, a considerable quantity of insect residue, known as insect frass, remains (Shi et al. 2022). Numerous studies have highlighted the capacity of this organic derived substance to increase soil fertility due to the presence of humic substances (Houben et al. 2020). Hence, insect frass is considered as a novel material that can potentially be utilized as PTE adsorbent for remediation efforts. When used for this purpose, insect frass can undergo pyrolysis resulting in biochar formation. The content of C in biochar can reduce PTE availability and positively influence microbial growth in soil (Yan et al. 2021). However, there is a lack of knowledge in up-to-the-date research whether non-pyrolyzed insect frass can affect the adsorption of PTEs in soil and if soil microbial community can influence this (Watson et al. 2021).

In order to understand and quantify sorption/desorption mechanisms, isotherm models can be utilized, among which the most often used are the Freundlich and Langmuir isotherms. These models describe the concentration of PTEs in the solid and liquid phases at constant temperature conditions (Golia et al. 2023). The Freundlich isotherm elucidates the partitioning of PTEs between soil and solution, especially in single-element systems and at low initial metal concentrations; hence, it is utilized for heterogeneous surfaces and nonlinear sorption. The distribution coefficient (*K*_d_) serves as an indicator of the transportability and retention of elements, with higher *K*_d_ values indicating stronger sorption (Shaheen et al. 2013). On the other hand, Langmuir isotherm describes an ideal monolayer adsorption process that was initially intended to describe homogeneous surfaces (Mu and Sun 2019). With this model, the maximum sorption capacity (*q*_max_) can be quantified, as the model allows the extrapolation of the observed data towards the highest sorption ability of an adsorbate (Wang & Guo 2023).

Even though there have been studies for estimating the sorption effectiveness of organic amendments to soil, there is limited research concerning the effect of frass under any batch sorption conditions. The only publication we could trace was that by Watson et al. (2021), which extracted heavy metals with dilute CaCl_2_ from soil after the addition of frass. Also, there have been numerous research efforts concerning the effects to soil parameters and plant growth of microbial-derived (e.g., of the *Bacillus* sp. genus) and humic/fulvic acid-derived biostimulants when applied to soil. However, to the best of our knowledge, such studies do not explore the possible effects that such biostimulants may have to the chemical behavior of metals such as Cd and Zn—thus, how frass and microbial- and organic-derived biostimulants alone and in combination may affect the chemical behavior of the soil matrix. Moreover, there is a void in the literature concerning the desorption of these metals from soils added with such amendments—thus, it is not known whether their possible positive effects in increasing soil retention towards metals will be long-lasting over time or whether metals will be readily desorbed after being initially retained. Furthermore, to the best of our knowledge, there is no information in the literature concerning the effect on metal sorption that these amendments may have in the soil matrix over time and if soil chemical behavior is altered after aging.

Thus, the current study aimed at identifying the sorption and desorption behavior of PTEs (Cd and Zn) in a pristine soil after its amendment with frass and microbial- and organic-derived biostimulants over time, i.e., in the beginning and at the end of a 30-day incubation of the mixtures (acting as sorbents). Thus, several amendments were tested as single additions, i.e., insect frass, and biostimulants derived from humic/fulvic substances and bacteria of the *Bacillus* genus; also their co-addition was tested (i.e., frass + humic/fulvic and frass + *Bacillus* sp.). The objectives of this study were to evaluate the interaction of these treatments in the sorption and desorption of PTEs in soil over time after an aging process. We see this as a crucial novel step into recognizing the optimal strategy for maximizing sorption with long-lasting effects and, if possible, minimizing desorption, in an effort of reduction the environmental bioavailability of PTEs and increasing soil health. The findings of this research are expected to contribute to the understanding of the mechanisms of PTE sorption in soil, introducing new insights into the use of novel methods for soil remediation.

## Materials and methods

### Experimental design

**Fig. 1:**
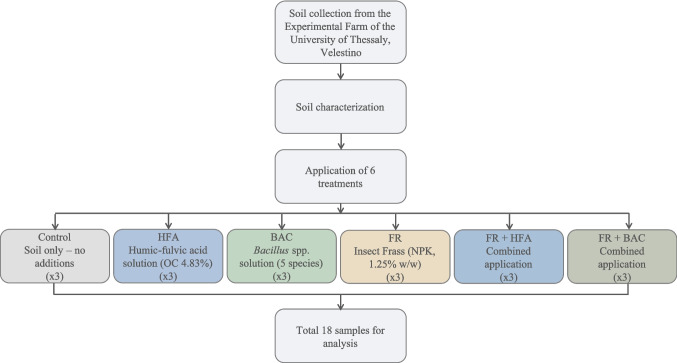
Overview of the experimental procedure

Α non-contaminated soil was collected from the Experimental Farm of the University of Thessaly in Velestino (22.756E, 39.395N). The soil was a sandy loam (45% sand and 16% clay), with pH 7.8, and organic carbon (OC) of 1.1%. The experiment consisted of six treatments (including the unamended control), each repeated three times, resulting in a total of 18 samples, as follows: (A) Control (soil with no additions). (B) Soil added with a solution of humic and fulvic acids (HFA) (extracted from leonardite in a 70:30 ratio; pH 8.53 and OC 4.83%). The HFA solution was diluted with distilled water and applied to soil by adding 180 mL of this biostimulant solution per kg soil. (C) Soil added with a biostimulant material consisting of a solid consortium of species of the *Bacillus* genus (*B. megaterium*,* B. altitudinis*, *B. subtilis*, *B. licheniformis*, and *B. methylotrophicus*) at a concentration of 109 cfu g^−1^ for each species (thereafter referred to as BAC). A weight of 0.6 g of the consortium was dissolved with 900 mL of distilled water, and the microbial biostimulant solution had a pH of 6.57 and OC 5.29%. Each replicate in this treatment received 150 mL of this solution per kg soil. (D) Soil added with insect frass (FR) derived from *Tenebrio Molitor*. Frass had a total nitrogen (N) of 4.98%, total phosphorus (P) 2.63%, total potassium (K) 1.65%, OC 45%, and pH 7.8, and was applied at a rate of 1.25% w/w (equivalent to 50 t ha^−1^ if taken at field level, calculating for a 30-cm incorporation zone and bulk density of that of typical medium-to-light-textured soil, such as our, i.e., 1.33 g cm^−3^). (E) Soil amended with the combination of frass and humic-fulvic acid solution (FR + HFA) at a same rate as the single applications. (F) Soil amended with the combination of frass and the *Bacillus* solution (FR + BAC) at a same rate as the single applications. The overall experimental procedure is summarized in the flowchart shown in Fig. 1.

The 18 soil mixtures (6 treatments × 3 replicates), which weighed ca. 500 g each, were put into plastic bags, watered to 2/3 of their field capacity, and placed in an incubation chamber at a constant temperature of 21 °C. The bags were kept open for better aeration and weight loss was monitored frequently and compensated with water addition. On day 1, samples from each bag were obtained and used in the batch sorption tests described below. The incubation had a duration of 30 days, and on the last day (day 30), another sample from each bag was obtained to be used for batch sorption test.

### Batch sorption experiment

To the 36 soil samples (18 from day 1 plus 18 from day 30), we conducted batch sorption tests. The samples were air-died and passed through a 2-mm sieve before a mass of 2 g of each was weighed into 50-mL centrifuge tubes, where also 20 mL of solution was added. The solution contained Cd and Zn as their chloride salts in systems containing both metals concurrently at a range of increasing initial concentrations (*C*_0_) of 0, 5, 10, 20, 40, 60, 80, and 100 mg L^−1^ with a background electrolyte of 10 mM CaCl_2_. The Cd and Zn concentrations were chosen to cover a wide range to examine soil sorption under varying levels of contamination. Lower concentrations represent environmentally relevant levels of metals typically found in contaminated soils, while higher concentrations allow for the determination of the soil’s maximum sorption capacity of metals and saturation. The tubes were shaken in a reciprocal shaker overnight (for at least 16 h), a sufficient amount of time to reach equilibrium, as judged by a series of preliminary tests (where equilibrium was attained in no more than 4 h). After that, the suspensions were filtered through slow filter paper and the concentrations of Cd and Zn in the equilibrium solution (the clear filtrate; equilibrium concentration annotated as *C*_e_ or simply *C*) were measured in a flame atomic absorption spectrophotometer (FAAS; Perkin Elmer A330). The concentration of metals adsorbed onto the solid (*q*) was measured by the difference *C*_0_ from that of *C* multiplied by a factor of 10 (the solution-to-solid ratio). To quantify and properly assess the sorption mechanisms of the studied metals, the two most commonly utilized isotherm models were used, Freundlich (*q* = *K*_F_*C*^*N*^) and Langmuir [*q* = *q*_max_*K*_*L*_*C*/(1 + *K*_L_*C*)], where *K*_F_, *N*, and *K*_L_ are constants computed from the curve fit. For the calculation of the sorption parameters, the models were first linearized as shown by Antoniadis and Golia (2015). To examine how well the models fitted the experimental data, the coefficient of determination (*R*^2^) was utilized, where a value close to 1 indicates a better fit; also the Derivative of Marquardt’s Percent Standard Deviation error function as indicated by Antoniadis and Golia (2015) in order to assess the accuracy of the data. Three indices were computed in order to assess metal sorption: *q*_100_ (the concentration of the metals sorbed at *C*_0_ = 100 mg L^−1^; based on the actual experimental data), *q*_max_ (computed based on the lineralized Langmuir isotherm), and *K*_d-50_ (the distribution coefficient, calculated as *q/C*, with units of L kg^−1^, computed from the Freundlich isotherm at *C*_0_ = 50 mg L^−1^).

In order to assess desorption, at the end of the equilibrium batch sorption test, the samples equilibrated with 100 mg L^−1^ of initial metal concentrations were extracted with DTPA, thereafter referred to as DTPA_100_ (as suggested by Antoniadis and Golia 2015). DTPA was used due to its strong ability to chelate metal ions, allowing their release from the surfaces of soil components. Its use aims to evaluate the reversibility of the adsorption process and the effectiveness of the applied soil amendments in binding and limiting PTE mobility in soil. The extraction was conducted for 2 h in a 1:2 solid-to-solution ratio using a DTPA-TEA-CaCl_2_ solution buffered at pH 7.3. Based on DTPA_100_, the percentage of desorption relative to that sorbed at 100 mg L^−1^ (thereafter referred to as Des%) was also calculated.

### Physicochemical property analyses

Furthermore, the 18 soil samples were measured as per Rowell (1994) for pH (1:2.5 H_2_O), electrical conductivity (EC; 1:5 H_2_O), and OC (Walkley and Black method of wet oxidation with surplus 0.166 M K_2_Cr_2_O_7_ and back-titrated with 0.5 M FeSO_4_). These three properties were selected to be measured because they could have been affected by the amendments. The measurements were conducted in both the samples of day 1 and of day 30; however, there were no differences between the two sampling times in each of the treatments; hence, data of day 30 are not shown.

### Statistical analysis and data quality control

In order to further evaluate the impacts of soil amendments on the retention and release of Cd and Zn, statistical analysis was performed on the sorption and desorption parameters both across different treatments and within the same treatment over time. All statistical tests were conducted using IBM SPSS Statistics 26. Data quality control was addressed by the systematic use of blank samples in each batch of analysis; all analyses were conducted in triplicates, and measurements were accepted when standard deviation was < 10%. Also soil reference materials for Cd and Zn (including an in-house reference material in each analysis batch plus the certified reference material BCR-141R-calcareous loam soil-extracted once) were used, and recovery was found to be satisfactory, ranging from 93 to 108%.

## Results

### Soil parameters

The addition to soil of the studied amendments (HFA, BAC, frass, and their combinations) led to varied effects in the measured key parameters (Table 1). pH had a non-significant change among treatments, ranging from 7.91 (FR) to 8.16 (HFA, BAC). The EC values exhibited significant variation, with the frass treatment (FR) demonstrating the highest value (581.67 μS cm^−1^), significantly higher than all the rest, which were not different among them. The lowest value was that of BAC (236.27 μS cm^−1^) and the control (253.43 μS cm^−1^). Regarding OC, it increased significantly from the control (1.10%) to the added biostimulants (HFA 1.29% and BAC 1.24%, both significantly higher than the control), and it further increased significantly in the treatments of added frass (FR 1.68%, FR + HFA 1.66%, and FR + BAC 1.65%).

**Table 1:** Soil parameters (pH, EC, and OC%) as influenced by the amendments

	pH	EC (μS cm^−1^)	OC%
C	8.10^a^	253.43^a^	1.10^a^
HFA	8.16^a^	252.57^a^	1.29^b^
BAC	8.16^a^	236.27^a^	1.24^b^
FR	7.91^a^	581.67^b^	1.68^c^
FR + HFA	8.04^a^	282.83^a^	1.66^c^
FR + BAC	8.08^a^	328.67^a^	1.65^c^
*Significance*	NS	**	***

Treatments are as follows: C (control), HFA (soil + humic and fulvic acid), BAC (soil + *Bacillus *sp.), FR (soil + frass), FR + HFA (soil + frass + humic and fulvic acid), FR + BAC (soil + frass + *Bacillus *sp.). Values are the mean of *n* = 3 replicates. Different small letters within columns denote significant differences between treatments based on the Duncan’s multiple range test (DMRT) (*p* < 0.05). The significance of differenceswithin each column are shown as follows: *significant at the level of *p* < 0.05; **significant at the level of *p* < 0.01; ***significant at the level of *p* < 0.001; *NS* non-significant

### Metal sorption and desorption

Regarding Cd distribution coefficient *K*_d-50_, it was not altered with the added amendments relative to the control, and similar was the case with Zn *K*_d-50_. However, Zn *K*_d-50_ was consistently and significantly higher than that of Cd. Over time, Cd *K*_d-50_ was found to have increased significantly at BAC (from 144.11 on day 1 to 196.70 L kg^−1^ on day 30) and FR (from 143.70 to 184.98 L kg^−1^), and similar was the case with Zn *K*_d-50_ over time at FR (from 940.09 to 1310.64 L kg^−1^) (Table 2). As for *q*_100_, it showed a significant increase at FR + HFA compared to the control on day 1, while Zn *q*_100_ did not have changes relative to the control. As in the case of *K*_d-50_, *q*_100_ was significantly higher for Zn than for Cd, although the differences were not as pronounced as those observed in *K*_d-50_. Over time Cd *q*_100_ increased at FR + HFA (840.98 to 890.04 me kg^−1^) and Zn *q*_100_ at FR (695.69 to 983.19 mg kg^−1^) and FR-BAC (965.00 to 982.67 mg kg^−1^). Regarding *q*_max_, similar to the previous two sorption indices, it exhibited the lowest value for Cd at C (944.44 mg kg^−1^), but it was found that the amendments did not increase Cd sorption beyond the control. As for Zn *q*_max_, it had an increasing trend compared to that of Cd, but differences were not as pronounced as in the previous two indices: only Zn *q*_100_ at FR and FR + HFA were significantly higher than the Cd *q*_100_ at control, while when compared among the Zn treatments, *q*_100_ exhibited no differences at all. Over time, there was no alteration in *q*_100_ in either metal at any treatments. Finally, Des% exhibited the lowest value at control for Cd, which had no significant differences from that of Zn at control. Cd Des% was increased in the various treatments compared to the control, but the differences were significant for the treatments FR and FR + BAC. The Zn Des% showed no differences when the various treatments were compared with each other. Interestingly, no significant alteration in Cd and Zn Des% was observed over time.

**Table 2:** Sorption parameters for Cd and Zn across all treatments on days 1 and 30

	*K*_d-50_ (L kg^−1^)		Sig	*q*_100 _(mg kg^−1^)		Sig	*q*_max _(mg kg^−1^)		Sig	Desorption (%)		Sig
	Day 1	Day 30		Day 1	Day 30		Day 1	Day 30		Day 1	Day 30	
Cd												
C	146.27^a^	188.80^a^	NS	823.51^a^	853.45^a^	NS	944.44^a^	690.58^a^	NS	2.08^a^	2.27^a^	NS
HFA	146.40^a^	151.49^a^	NS	853.04^ab^	869.25^ab^	NS	1000^ab^	685.19^a^	NS	5.01^abc^	5.35^b^	NS
BAC	144.11^a^	196.70^a^	**	857.19^ab^	876.32^ab^	NS	1000^ab^	558.46^a^	NS	4.71^abc^	5.41^b^	NS
FR	143.70^a^	184.98^a^	*	840.98^ab^	890.04^b^	*	1006.73^ab^	867.52^ab^	NS	5.74^bc^	5.63^b^	NS
FR + HFA	170.65^a^	169.40^a^	NS	872.99^b^	860.52^ab^	NS	1074.07^ab^	734.67^ab^	NS	4.60^abc^	5.79^b^	NS
FR + BAC	135.44^a^	153.70^a^	NS	835.99^ab^	885.05^b^	NS	944.44^a^	749.46^ab^	NS	6.26^c^	5.92^b^	NS
Zn												
C	1163.46^b^	1718.52^b^	NS	963.11^c^	967.06^c^	NS	1157.41^b^	1074.07^abc^	NS	3.16^ab^	1.87^a^	NS
HFA	827.92^b^	1705.02^b^	NS	960.54^c^	973.75^c^	NS	1157.41^b^	1043.45^abc^	NS	2.61^a^	2.89^a^	NS
BAC	910.72^b^	1410.54^ab^	NS	962.09^c^	969.46^c^	NS	1111.11^ab^	1074.07^abc^	NS	2.46^a^	3.17^a^	NS
FR	940.09^b^	1310.64^ab^	*	965.69^c^	983.19^c^	**	1120.37^b^	1263.23^bc^	NS	3.04^ab^	3.07^a^	NS
FR + HFA	1175.50^b^	2137.64^b^	NS	977.70^c^	975.30^c^	NS	1322.75^c^	1537.04^c^	NS	2.38^a^	2.97^a^	NS
FR + BAC	1027.88^b^	1770.80^b^	NS	965.00^c^	982.67^c^	**	1074.07^ab^	939.39^ab^	NS	3.23^ab^	2.88^a^	NS
Sig	***	***		***	***		**	*		**	***	

Treatments are as follows: C (control), HFA (soil + humic and fulvic acid), BAC (soil + *Bacillus *sp.), FR (soil + frass), FR + HFA (soil + frass + humic and fulvic acid), FR + BAC (soil + frass + *Bacillus *sp.). Values are the mean of *n* = 3 replicates. Different small letters within columns denote significant differences between treatments based on the Duncan’s multiple range test (DMRT) (*p* < 0.05). The significance of differences over time (between days, i.e., day 1 vs. day 30) and within each column are shown as follows: *significant at the level of *p* < 0.05; **significant at the level of *p* < 0.01; ***significant at the level of *p* < 0.001; *NS* non-significant

**Fig. 2:**
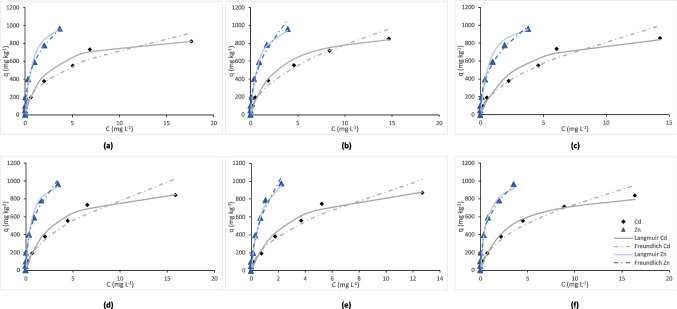
Cd and Zn sorption isotherms at day 1 in all treatments: **a** control, **b** HFA, **c** BAC, **d** FR, **e** FR + HFA, **f** FR + BAC. Treatments are as follows: C (control), HFA (soil + humic and fulvic acid), BAC (soil + *Bacillus *sp.), FR (soil + frass), FR + HFA (soil + frass + humic and fulvic acid), FR + BAC (soil + frass + *Bacillus *sp.)

**Fig. 3:**
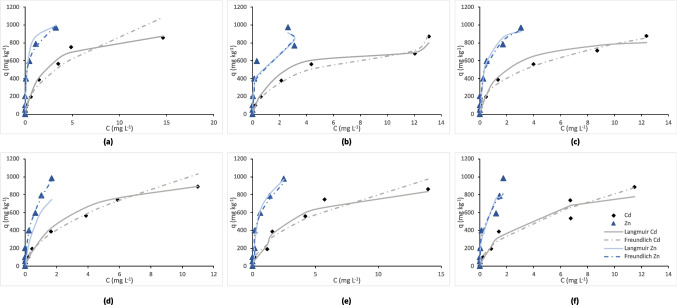
Cd and Zn sorption isotherms at day 30 in all treatments: **a** control, **b** HFA, **c** BAC, **d** FR, **e** FR + HFA, **f** FR + BAC. Treatments are as follows: C (control), HFA (soil + humic and fulvic acid), BAC (soil + *Bacillus *sp.), FR (soil + frass), FR + HFA (soil + frass + humic and fulvic acid), FR + BAC (soil + frass + *Bacillus *sp.)

As for the adsorption isotherms (Fig. 2 for the sorption of the two metals on day 1, Fig. 3 for their sorption on day 30), they demonstrate the relationship between the equilibrium concentration of the studied metals (Cd and Zn) in solution (*C*, mg L^−1^) and the concentration sorbed per unit mass of soil (*q*, mg kg^−1^). Both Cd and Zn showed the same adsorption curve shape characteristic of the L-type curves, based on the Giles classification of adsorption isotherms. The experimental data were fitted to both the Langmuir and Freundlich isotherm models satisfactorily; however, Langmuir exhibited a higher coefficient of determination (*R*^2^ range was 0.801–0.996) and lower error function values compared to Freundlich (*R*^2^ range was 0.734–0.993; Supplementary Table 1). Moreover, based on the comparative analysis of the adsorption curves of the two studied metals, significant differences in their behavior were revealed: Zn had a steeper sorption curve compared to Cd, hence higher affinity to soil colloids at lower concentrations. On the other hand, Cd showed a more gradual adsorption trend, suggesting a lower binding strength compared to Zn.

## Discussion

Among the measured soil properties (Table 1), pH indicated no significant alterations in the treatments compared to the control, a finding that signifies the fact that any changes observed in the metal chemical behavior cannot have been induced due to pH, which otherwise is usually the single most dominant factor influencing metal sorption and thus metal bioavailability. OC was found to be significantly higher in all additions compared to the control. The enhancement was especially pronounced in the frass addition treatments (FR, FR + HFA, and FR + BAC), with the difference being as much as 0.5% of added OC w/w soil (from 1.10% at C to 1.68% at FR). This would be equivalent, if extrapolated at field scale, to 22.5 t of added OC ha^−1^, an enormous addition that would increase soil health and enhance soil biodiversity and biological activity. This increase is interestingly as expected for the addition of frass at 1.25% w/w and with an OC content of 45%. Hence, this property alone (added organic matter to soil) was the decisive factor that influenced the chemical behavior of the studied metals in their retention by soil. Also the frass-added (as well as the humic/fulvic and *Bacillus* biostimulants-added) OC must have induced significant biological activity to soil over the 30-day incubation period, accelerating thus soil aging and affecting further metal chemical fate and activity.

As evidenced by Figs. 2 and 3, Zn was adsorbed at a higher rate compared to Cd, a finding also concurring with the computed sorption indices discussed below. This may have happened due to the ionic radius and the competitive nature of the sorption process in this study. Despite the fact that both Zn and Cd are divalent cations, Zn is characterized by smaller ionic radius, size, and density, and this can lead to stronger binding onto soil particles due to the higher charge-to-radius ratio, which increases sorption selectivity. This is also proved by the fact that Zn outcompetes Cd for the limited sorption sites of soil colloids, exhibiting a maximum adsorption capacity (*q*_max_) of as high as 1537.04 mg kg^−1^ at FR + HFA in day 30. The obtained experimental data fitted better the Langmuir isotherm over that of Freundlich, as judged by the higher *R*^2^ and lower error function values (Supplementary Table 1). In general, the Langmuir model suggests a monolayer adsorption, indicating that all adsorbent sites occupy only one molecule, each of which forms a single layer on the surface. According to the approach of this model, once all sites are occupied, no further adsorption can occur. On the other hand, the Freundlich model assumes a multilayer adsorption, indicating that adsorbates can accumulate in more than one layer after the first layer has been formed. According to this approach, adsorption sites are not necessarily uniform, meaning that the surface sites exhibit multiple affinities for the adsorbent (Kim et al. 2025). The same results as in our study were reported by Rassaei et al. (2020), who studied the impact of Zn competition on Cd retention in different types of soil by performing a sequential fractionation. They found that ultimately Zn competed and reduced the concentration of Cd associated with the Fe/Mn oxides fraction due to Zn sorption competition regarding the oxide surfaces. Also, Zn reduced Cd concentration associated with the carbonate and organic matter fractions as a result of its precipitation; this occurred with the formation of insoluble carbonate metal species and metal binding with organic ligands for the formation of stable organometallic complexes. This same effect happened also in our case where Zn exhibited more rapid adsorption, reaching saturation more quickly even at lower concentrations across all applied amendments (Figs. 2 and 3). Our findings seem also to concur with those of Yang et al. (2021), who predicted metal adsorption in soils from data extracted from 150 Journal publications. They found that the overall metal adsorption capacity followed the order Pb > Zn > Cr > Cu > Cd > Ni, indicating thus a predominance of Zn sorption over that of Cd, as also observed here. In addition, they found that Zn exhibited the lowest variability, meaning that its adsorption capacity was relatively consistent across different soil types and regions.

The computed sorption indices (Table 2) exhibited significant variations in Cd retention and desorption among the treatments, underscoring the importance of employing the suitable amendment for the management of soil contamination. Distribution coefficient, *K*_d-50_, expresses the mobility of an element in soil—high metal *K*_d-50_ indicates strong sorption onto soil surfaces and thus low metal solubility, a likely indication of low environmental bioavailability. It is thus a key index for evaluating the mobility of PTEs through adsorption phenomena (Antoniadis et al. 2007). The observed significant increase in the Cd *K*_d-50_ on day 30 compared to day 1 in the BAC and FR treatments indicates an enhancement in Cd retention over time, which is likely due to mechanisms associated with the biological interaction of soil colloids with the added bacteria (BAC) and specific chemical properties of frass (FR). Concerning the former, the association of cationic PTEs with the soil microbial community is a complex process that involves various mechanisms, such as extracellular biosorption and intracellular bioaccumulation (Chi et al. 2020). More specifically, metal retention is possible via ion exchange, physical trapping, and formation of functional groups onto cell surface protons (-COOH, -NH), which help to enhance binding of Cd to the bacterial surface (Alotaibi et al. 2021; Chi et al. 2020). Furthermore, PTEs can be incorporated into cellular organelles and bind to metallothioneins through the bacterial metabolic processes, reducing its concentration in the environment (Yuan et al. 2019). This combination of intracellular and extracellular processes seems plausible only if sufficient aging time is allowed, so that full microbial functionality may be activated in watered and incubated soils in constant aerated and temperature conditions (in our case, at 21 °C). This indeed seems to be the situation in our trial, where soils were incubated after the addition of the *Bacillus* biological material so that microbial activity may have sufficient time to interact chemically and influence the fate of metals with soil aging. In a study by Charan et al. (2024), *Bacillus xiamenensis* showed a selective adsorption capacity of PTEs in a multi-metal system, indicating that microbial biomass interacted with metals with different efficiencies. It is reasonable to assume that similar selective interactions do affect the adsorption capacity in the soil-microorganism system. As for the adsorption of Zn, it increased at FR from day 1 to day 30, similar to Cd, indicating an enhancement in Zn retention over time, as demonstrated by the *K*_d-50_ value. In the case of Zn, the effect of *Bacillus* alone was not a sufficient factor to enhance Zn retention. Frass seems to have triggered the activity of the autochthonous soil microbial community, which was decisive in playing a role in Zn retention. On the other hand, frass-borne organic matter increased the available retention sites in soil, enhancing thus the overall soil functionality concerning ion exchange. Insect frass is known to be in abundance of carboxylic (-COOH) and phenolic (-OH) active groups, as part of its organic matter, which can bind PTEs and enhance the adsorption capacity and limit metal mobility (Kim et al. 2025). This is especially the case in usual neutral-to-alkaline soil pH values, such as was the case in our soil, where organic matter has higher cation exchange capacity values compared to that in acidic pH values. This happens because these active groups are typically deprotonated, as the proton equilibrium between these groups and the soil solution is shifted towards the solution, in order to counter-balance the low proton activity (hence the high pH) in soil solution (Antoniadis et al. 2017).

The increase in metal retention, as judged by *K*_d-50_, was not fully observed when *q*_max_ was taken into consideration, as the only significant difference of this index among treatments was found for FR + BAC relative to the control for Zn (and no significant changes in any of the Cd treatments). All the same, no significant differences seemed to be induced with soil aging. This is an indication of the fact that active adsorption sites of the soil matrix may have not be altered, or may even be saturated or blocked by organic substances in the long term as suggested by Chen et al. (2022). It may, thus, be the case that when added organic materials are fully humified and incorporated into the soil matrix, the effects may be more pronounced; however, such effects were too early to be observed in our test, as humification is a process that requires longer periods of time than those studied here. It is widely accepted that the quantity of active adsorption sites on the surface of an adsorbent influences the total amount of PTEs that are being sorbed (Zhang et al. 2021). Moreover, *q*_max_ is an index of the ultimate sorption capacity of soil, which is not limitless, and the high initial concentration tested here (*C*_0_ = 100 mg L^−1^) seems to have led to a plateau for the studied metals even in the unamended soil. This is especially the case in our study where the competitive systems tested here (where Cd and Zn coexisted—in our attempt to simulate real multi-metal soil contamination cases) must have further suppressed the soil’s sorption capacity **(**Uddh-Söderberg et al. 2024).

Similar to our findings, Kim et al. (2025) observed that Cd sorption by mealworm frass increased with Cd concentration up to a saturation limit at concentration above 100 mg L^−1^. This suggests that frass has the capacity to sorb Cd up to certain limit of active sites. On the other hand, *K*_d-50_ is an index reflecting metal chemical behavior in the mid-range of the initial concentration, representing thus more closely non-extreme environmental cases of metal contamination. Thus conclusions and field extrapolations are likely safer when based on this index rather that when assessed by the highest soil sorption capacity (*q*_max_).

Although this was the case with *q*_max_, the index of *q*_100_, which measures actual observed sorption data, exhibited a different picture concerning the effect of the added organic materials to soil: Cd *q*_100_ at FR + HFA was significantly higher compared to the control, signifying thus the importance of the active humic substances to soil in combination with frass. Indeed frass, due to its inherently low C/N ratio (Hwang et al. 2022), can be fast decomposed and readily produce organic substances at the molecular weight range of the well-humified soil humic substances (i.e., in the order of magnitude of a few tens of thousands g mol^−1^) (Shi et al. 2025). Such frass-induced organic substances, assisted by those added directly to soil with the fulvic/humic biostimulant solution (at HFA), may have created a favorable environment for the enhanced retention of Cd, a metal known for its affinity with organic substances to a greater extent than that of Zn (Liu et al. 2024). This effect of frass was so important that was observed in its behavior over time: Cd *q*_100_ was significantly increased at FR in day 30 relative to day 1, showing that soil aging with frass was beneficial concerning its overall retention capacity. This also comes as a confirmation of the fast decomposing nature of frass explained earlier. These findings show that frass and its combinations with the other tested materials are more stable over time and effective solution for enhancing Cd retention by soil. As for Zn, no significant alterations in *q*_100_ were found in its sorption in the amended treatments compared to the unamended control, a likely outcome of the competitive conditions in our multi-metal systems tested here. However, at FR, Zn *q*_100_ exhibited a significant increase in day 30 compared to that in day 1, concurring thus with the increase observed for Cd. This remarkable increase in the FR treatment indicates that frass may have had certain properties that enhanced its ability over the time, likely similar to those discussed earlier. Same was the case with FR + BAC, signifying the beneficial effect of activated microbial soil community induced by the added *Bacillus* strains, as also discussed earlier.

In a further attempt to assess Cd and Zn fate, we measured their desorption and recorded it as a percentage of that extracted from the soil at the added initial concentration of 100 mg L^−1^ over the actual *q*_100_ value. Cd desorption was not different from that of Zn in the unamended soil in both days 0 and 30, indicating their stability once they are bound by the soil matrix. Cd desorption at FR and FR + BAC was significantly elevated relative to the Cd desorption at control. This is an indication of the fact that added organic materials in soil are far from the point of reaching maturity in soil and of being fully incorporated and humified in the soil system. This means that over the study time, the added materials behaved in the colloidal level as distinct phases, which are more prone into altering their metal sorption behavior than soil colloids are. However, this was a case for Cd only, as Zn desorption was not found to have increased in the amended treatments relative to the Zn control both in days 1 and 30. This concurs with our findings discussed earlier, i.e., that Zn was more strongly retained than Cd, which was rather poorly sorbed. This also concurred with the findings by Ghosh and Kartha (2025). However, even in the case of Cd, its desorption was not accelerated over time, a promising finding concerning the eventual stability of the added organic materials concerning their chemical behavior towards Cd and Zn. Although this study did not perform speciation analysis (the assessment of PTE fractions such as organic-bound, exchangeable and residual fractions, which influence PTE mobility and availability), this approach could provide insights regarding the mechanisms of metal binding and release however, such analysis is recommended for future research.

## Conclusions

This study showed that the adsorption of Cd and Zn was satisfactorily explained employing both Langmuir and Freundlich models in all treatments; however, the Langmuir isotherm had a marginal advantage over that of Freundlich. The elevated *K*_d-50_ values for Cd at FR and BAC in day 30 over that in day 1 highlight the effectiveness of frass and the microbial-based biostimulant to enhance Cd binding over time. Our findings also emphasize the performance of both insect frass and its combination with *Bacillus* in stabilizing both tested metals with a variety of sorption mechanisms. These mechanisms are likely related to active functional groups for the organic material, mainly carboxylic and phenolic, which are readily available for association with cationic metals such as Cd and Zn due to their deprotonated nature in the pH range found in our treatments. Mechanisms are also likely related to intracellular and extracellular microbial processes resulting in metal binding. Cd desorption was higher at FR and FR + BAC relative to the control, a finding showcasing the immaturity of these materials into being fully incorporated into the soil matrix. However, the fact that no further increase in desorption was observed over time is a rather promising outcome, signifying the possibility of these materials reaching maturity into soil over a relatively brief amount of time, unlike other organic materials. We conclude that the tested amendments functioning as soil improvers, alone or combined, may act in a beneficial way into reducing the environmental risks of PTE availability, while their aging after being added to soil will lead to their stability. We see this work as an initial step into a necessary in-depth study of the nature and behavior of the tested novel (and thus still largely unknown) materials concerning the chemical fate and behavior of environmental contaminants. Future work should thus employ advanced analytical techniques that would further explore the exact mechanisms governing the chemical behavior of metals in soil in the presence of these novel materials, especially in soil aging processes over longer periods of time.

## Supplementary Information

Below is the link to the electronic supplementary material.

Supplementary Material 1 (DOCX 20.7 KB)

## Data Availability

Data will become available upon reasonable request.
