# Supplementary material for: Cadmium and zinc sorption and desorption in soil: the impact of humic-fulvic acids, Bacillus sp., insect frass, and soil aging
Source: Environ Sci Pollut Res Int. 2025 Jul 8;32(29):17856–67. doi: 10.1007/s11356-025-36699-4 (PMC12325448; doi:10.1007/s11356-025-36699-4)
Supplement: Supplementary file 1 — Supplementary Material 1 (DOCX 20.7 KB) [file 11356_2025_36699_MOESM1_ESM.docx]

|  |  | | **Langmuir** | | | | | | **Freundlich** | | | | | | | |
| --- | --- | --- | --- | --- | --- | --- | --- | --- | --- | --- | --- | --- | --- | --- | --- | --- |
|  | **DTPA_100_**  mg kg^-1^ | | ***K_L_***  L mg^-1^ | | **MPSD** | | ***R^2^*** | | ***K_F_***  mg Kg^-1^ | | ***N*** | | **MPSD** | | ***R^2^*** | |
| **Cd** | **Day1** | **Day30** | **Day1** | **Day30** | **Day1** | **Day30** | **Day1** | **Day30** | **Day1** | **Day30** | **Day1** | **Day30** | **Day1** | **Day30** | **Day1** | **Day30** |
| C | 57.33 | 19.16 | 0.461 | 1.089 | 1.449 | 1.158 | 0.972 | 0.965 | 268.7 | 275.0 | 0.44 | 0.51 | 1.126 | 1.183 | 0.965 | 0.950 |
| HFA | 42.70 | 46.27 | 0.362 | 0.897 | 1.090 | 1.291 | 0.988 | 0.970 | 239.5 | 250.8 | 0.52 | 0.50 | 1.102 | 1.186 | 0.972 | 0.950 |
| BAC | 40.54 | 47.27 | 0.368 | 5.248 | 1.160 | 1.473 | 0.975 | 0.921 | 246.2 | 308.0 | 0.53 | 0.41 | 1.082 | 1.123 | 0.977 | 0.969 |
| FR | 48.44 | 50.10 | 0.362 | 0.622 | 1.133 | 1.112 | 0.985 | 0.989 | 237.2 | 276.4 | 0.53 | 0.55 | 1.106 | 1.104 | 0.971 | 0.981 |
| FR+HFA | 40.04 | 49.77 | 0.364 | 1.207 | 1.313 | 1.671 | 0.963 | 0.925 | 262.1 | 262.2 | 0.53 | 0.50 | 1.192 | 1.622 | 0.949 | 0.895 |
| FR+BAC | 52,10 | 52,43 | 0.362 | 0.676 | 1.129 | 1.315 | 0.979 | 0.957 | 230.0 | 240.0 | 0.51 | 0.53 | 1.110 | 1.259 | 0.968 | 0.927 |
| **Zn** |  |  |  |  |  |  |  |  |  |  |  |  |  |  |  |  |
| C | 30.45 | 18.01 | 1.517 | 4.167 | 2.776 | 3.082 | 0.978 | 0.996 | 595.3 | 738.1 | 0.38 | 0.26 | 2.683 | 3.006 | 0.978 | 0.987 |
| HFA | 25.10 | 28.07 | 1.267 | 16.478 | 2.375 | 4.557 | 0.994 | 0.988 | 570.7 | 689.8 | 0.46 | 0.31 | 2.432 | 3.383 | 0.924 | 0.966 |
| BAC | 23.72 | 30.66 | 1.725 | 2.333 | 2.265 | 3.082 | 0.979 | 0.971 | 586.6 | 683.5 | 0.39 | 0.34 | 2.047 | 3.006 | 0.970 | 0.930 |
| FR | 29.34 | 30.22 | 1.814 | 1.022 | 1.990 | 3.627 | 0.981 | 0.957 | 616.2 | 759.2 | 0.41 | 0.46 | 1.789 | 2.678 | 0.986 | 0.993 |
| FR+HFA | 23.27 | 28.94 | 1.322 | 2.538 | 2.208 | 3.127 | 0.943 | 0.827 | 692.2 | 689.9 | 0.54 | 0.37 | 2.279 | 3.185 | 0.866 | 0.830 |
| FR+BAC | 31.17 | 28.32 | 1.695 | 3.889 | 2.856 | 2.221 | 0.971 | 0.801 | 619.5 | 689.3 | 0.34 | 0.33 | 2.691 | 2.912 | 0.962 | 0.734 |

**Supplementary Table 1.** Sorption parameters for Cd and Zn according to the Langmuir and Freundlich isotherm models.

^1^ Treatments are as follows: C (Control), HFA (Soil + Humic and Fulvic Acid), BAC (Soil + *Bacillus sp.*), FR (Soil + Frass), FR + HFA (Soil + Frass + Humic and Fulvic Acid), FR + BAC (Soil + Frass + *Bacillus sp.*).

DTPA_100_ Extraction with DTPA of metals sorbed by soil at *C*_0_=100 mg L^-1^

MPSD Marquardt’s percent standard deviation
